# Community and familial dynamics influencing risk behavior for HIV acquisition among adolescent girls and young women in Uganda: Qualitative analysis using Protective Motivation Theory

**DOI:** 10.1371/journal.pone.0301311

**Published:** 2025-01-24

**Authors:** Rose Apondi, Hilde Bastiaens, Christiana Nöstlinger, Jennifer Galbraith, Tiffiany M. Aholou, Amy Medley, Rhoda K. Wanyenze, Anna C. Awor, David M. Serwadda, George Aluzimbi, Juliet Cheptoris, Moses Ogwal, Neema Nakyanjo, Pragna Patel

**Affiliations:** 1 Division of Global HIV & TB, US Centers for Disease Control and Prevention (CDC), Kampala, Uganda; 2 Department of Family Medicine and Population Health, University of Antwerp, Antwerp, Belgium; 3 Department of Public Health Antwerp, Institute of Tropical Medicine, Antwerp, Belgium; 4 Division of Global HIV & TB, US Centers for Disease Control and Prevention, Atlanta, GA, United States of America; 5 Makerere University, School of Public Health, Kampala, Uganda; 6 Ministry of Health, Kampala, Uganda; 7 Rakai Health Sciences Program, Kampala, Uganda; Makerere University, UGANDA

## Abstract

**Background:**

In Uganda, adolescent girls’, and young women’s (AGYW-15-24 years) current HIV prevalence is fourfold compared with their male counterparts due to compounded social, economic, and environmental factors. Using the Protective Motivation Theory (PMT), we explored HIV-acquisition risk sources and perceived protective factors from AGYW and caregivers’ perspective.

**Materials and methods:**

During 2018, we conducted a qualitative study guided by PMT to explore factors influencing HIV acquisition among AGYW. We purposively sampled two groups of key informants, AGYW at high-risk for HIV acquisition (uninfected) and AGYW living with HIV, varied by age and place of residence (urban/rural). We conducted 34 focus group discussions with AGYW, nine with AGYW parents, and 25 key informant interviews. Data were analyzed using the framework method based on the PMT and developed from participants’ narratives.

**Results:**

AGYW were knowledgeable about HIV, HIV acquisition risk factors, and HIV prevention interventions. Nonetheless, few AGYW knew about pre-exposure prophylaxis (PrEP). Imbalance in power relations between the genders explained inability of AGYW making safe healthy decisions, with social norms giving men power over women. Parents modelling positively influenced HIV risk behavior. Many AGYW viewed staying in school a protective factor both while at school and further for life. AGYW identified alcohol use, desire for material possessions, discounting HIV disease severity, social norms, and poverty as barriers to engaging in self-protective behaviors. Several AGYW believed that access to AGYW-focused programs would facilitate healthy sex-positive, protective behaviors.

**Discussion:**

While PMT focuses on individual factors confirmed by our findings, we found HIV risk behavior to be influenced by complex contextual factors including poverty, gender inequality and cultural norms. Distinct HIV risk factors among AGYW require policy and comprehensive targeted interventions addressing violence, alcohol consumption, increased economic opportunities, educational opportunities, safe-sex practices, and PrEP scale-up which may prevent HIV in AGYW and facilitate HIV epidemic control.

## Introduction

The most recent Uganda Population HIV Impact Assessment (UPHIA 2020) found that HIV prevalence was 2.9% among adolescent girls and young women (AGYW) aged 15–24 years compared with 0.8% among their male counterparts [1]. Poverty, gender inequality, low education level, early sexual debut, early pregnancy, gender-based violence, gender norms, inability to negotiate safer sex and sexual risk behaviors are associated with elevated HIV acquisition risk for AGYW [2–4]. One consistent protective factor for several negative health and social outcomes, including HIV, is having a positive parental relationship [5]. Additionally, creating intentional AGYW safe spaces can directly affect individual agency and ability for self-protective behaviors [4, 6]. Community engagement, like church membership or youth-friendly centers, sports facilities and spending time with family and youth leadership in programs were found to be associated with reduced HIV acquisition [7, 8]. Family and peer influences can therefore impact decisions to engage in self-protecting sexual behaviors [6].

Behavior change models recommend interventions focused on modifiable constructs with the greatest probability of increasing protective behaviors while inhibiting risky behaviors [9]. One evidence-based model, the Protection Motivation Theory (PMT), outlines four key elements: information source, threat appraisal, coping appraisal (which encompasses response efficacy and self-efficacy) and intentions to action [10]. PMT has informed the development of several adolescent HIV behavioral interventions [9, 11]. From the PMT, environmental and personal factors affect one’s choice for self-protective behaviors, considering either intrinsic (personal pleasure) or extrinsic rewards (social approval) and the perceived severity of and personal vulnerability to the threat [10].

The PMT allows for the examination of several developmentally relevant variables (e.g., adolescent perceptions of invulnerability and changing relationships with parents and peers to gain autonomy) and accommodating economic, historical, and cultural influences in the community [12]. Describing contextual factors that influence HIV risk behaviors among AGYW through qualitative methods can provide a richer framework, given that many studies have mostly quantified the HIV risk among AGYW [11, 13]. Yet, few studies applied a systematic approach to identity factors affecting HIV risk for AGYW using the PMT. Such research was mainly conducted in the USA and no studies are available for in Eastern Africa using this theoretical model [14]. Using the PMT, we sought to explore the contextual factors influencing HIV-acquisition risk sources and perceived protective factors from AGYW and caregivers’ perspective to inform targeted high-risk AGYW HIV prevention programs. We further aimed to examine the roles of peers and parents in influencing risk behaviors of AGYW from the perspective of AGYW themselves and their parents.

## Materials and methods

### Study design and setting

We conducted a qualitative study with combined deductive and inductive approaches to explore familial and community factors influencing HIV acquisition among AGYW [15, 16]. Data were collected through 34 focus group discussions (FGDs) and 25 key informant interviews (KIIs) (Table 1). A total of 34 focus group discussions were held including 9 with parents/guardians of AGYW, 8 with AGYW living with HIV, and 17 with AGYW at high risk of HIV acquisition. Participants were selected from districts in the greater Masaka and Kampala areas. The districts were purposively selected based on factors associated with high HIV acquisition risk among AGYW, including higher than 7% HIV prevalence, average age of first birth, number of young women with early sexual debut (defined as sex <15 years), and percentage of women reporting first sex as coerced. The US President’s Emergency Plan for AIDS Relief (PEPFAR) DREAMS (Determined, Resilient, Empowered, AIDS-free, Mentored, and Safe) program is implemented in districts in the greater Masaka region. DREAMS is a multipronged HIV-prevention approach for AGYW, sexual partners, families, and communities that include parenting/caregiver programs, cash transfers, educational subsidies, and HIV prevention interventions [4].

**Table 1:** Data analysis.

**Familiarization**	All researchers became familiar with the data by thoroughly reading the transcripts.
**Identification of a thematic framework**	Three researchers independently coded the same three transcripts before meeting to discuss key themes and constructing an initial coding framework. Using this framework, two researchers coded three additional transcripts each before discussing and refining the work. The process was repeated until no new themes were generated, and the final thematic framework was agreed upon. We used the PMT as a guidance to map the themes.
**Indexing**	The thematic framework was systematically applied to all transcripts. The researchers then organized codes which were valuable for organizing the research question into categories reflecting prominent themes within the dataset. The themes were then developed around the constructs of the Protection Motivation Theory
**Charting**	A matrix was created for each theme by abstracting, summarizing, and charting data from each transcript and each code within that theme.
**Mapping and interpretation**	Thematic analysis was carried out on the managed dataset by reviewing the matrices and making connections within and between codes and cases. This process was influenced by the original research objectives and by concepts generated inductively from the transcripts.

During June and July 2018, we purposively sampled two AGYW key informant groups: AGYW at high risk for HIV acquisition and AGYW living with HIV, ensuring representation by age (15–17, 18–24) and residence (urban/Kampala, rural/Greater Masaka) [17]. We used similar criteria to conduct FGDs with parents/guardians of AGYW at high risk for and living with HIV. We outline all factors considered in the purposeful sampling for the FGDs and KIIs in S1 Appendix.

### Data collection

Data were collected by trained qualitative interviewers using semi-structured interview guides. The team of interviewers were half female and half male, all with experience qualitative interviewing skills, all social workers while the supervisor was a social scientist. As part of the creating a safe space, emphasizing confidentiality and trust, the team started with self-introductions, clarifying their credentials, occupation, and training.

Based on the PMT, separate guides were developed for AGYW with and without HIV and their parents/guardians and for the two data collection techniques. These guides were developed in English, translated into Luganda, back translated into English, and piloted with mock participants for accurate translation. All discussions were held in the local Luganda language and audio recorded to create a verbatim transcript for each FGD and KII.

### Data analysis

We used the framework method for data analysis, which is an adequate approach for data collected in line with the overarching PMT framework. It is an excellent tool for supporting systematic managing and mapping of the data [18]. This allowed for extraction of detailed, experiential material from the data to examine in the context of the application and proposed expansion of PMT [19].

Data were coded in NVivo V1 (QSR International, Melbourne, Australia) using both deductive and inductive coding by developing themes from both the participants’ narratives using the PMT as guidance [20, 21]. A codebook was developed as outlined in Table 1. Three analysts, all behavioral scientists with experience in qualitative research (RA, TMA, AM) coded transcripts and regularly met to discuss any discrepancies allowing further transcript exploration, constant discussion of deviant cases, and agreement on recurring themes.

#### Ethics and consent procedures

Ethics approval was given by the Uganda National Council of Science and Technology and Institutional Review Boards (#4443) at the Makerere School of Public Health (#509) and the US Centers for Disease Control and Prevention (CDC). Written informed consent, including audio recording, was obtained from all participants. Additionally, all participants under age 18 years provided written informed assent and caregiver consent, excepting emancipated minors. According to the “National Guidelines for Research Involving Humans as Research Participants”, published March 2007 by the Uganda National Council for Science and Technology (http://www.uncst.go.ug, excerpt attached) “…emancipated minors are individuals below the age of majority who are pregnant, married, have a child, or cater for their own livelihood” and “…and may independently provide informed consent…” (p. 33). Confidentiality assurances were given during the assent/consent process. Names and other personal identifiers were removed from transcripts before they were analyzed.

## Results

### Participant characteristics

Table 2 shows participant characteristics by age category for the KIIs and FGDs. Among the adolescent girls aged 15–17, 38% were living with HIV compared to 42% of the young women aged 18–24. About 46% of the participants 15–17 years old reported that they attended school compared to 50% among the young women aged 18–24 years.

**Table 2:** A. Participant characteristics of Adolescent Girls and Young Women key informant interviews. B: Participant characteristics of focus group discussions for both Adolescent Girls and Young Women and parents/guardians (N = 34).

A			
**Age of participants (n)**	**All**	**15–17 (n = 13)**	**18–24 (n = 12)**
**Setting**			
Urban- Kampala	13	6	7
Rural-Rakai	12	7	5
**School Status**			
In School	12	6	6
Out of School	12	7	6
**HIV Status**			
Uninfected	15	8	7
Living with HIV	10	5	5
**Marital Status- AGYW**			
Single	23	13	10
Married	1	0	1
Co-habiting	1	1	0
**Religion**			
Protestant	5	2	3
Catholic	14	8	6
Muslim	3	3	0
Pentecostal	3	1	2
**Living Conditions**			
Lives with parents	10	6	4
Lives with partner	2	1	1
Lives alone	2	1	1
Lives with relatives	2	4	0
Homeless	2	1	1
Not reported	4	2	2
Friends	3	2	1
**Has a sexual partner (last 12 months)**			
Yes	20	12	8
No	4	1	3
**B**			
	**Positive**	**Negative**	**# participants**
**Kampala**			**121**
Parents/guardians of AGYW	2	2	43
AGYW	5	8	78
**Rakai**			**116**
Parents/guardians of AGYW	2	3	36
AGYW	3	9	80
**Total number of FGDs**	**12**	**22**	**237**

#### Contextualized PMT describing the AGYW HIV risk

**Fig 1:**
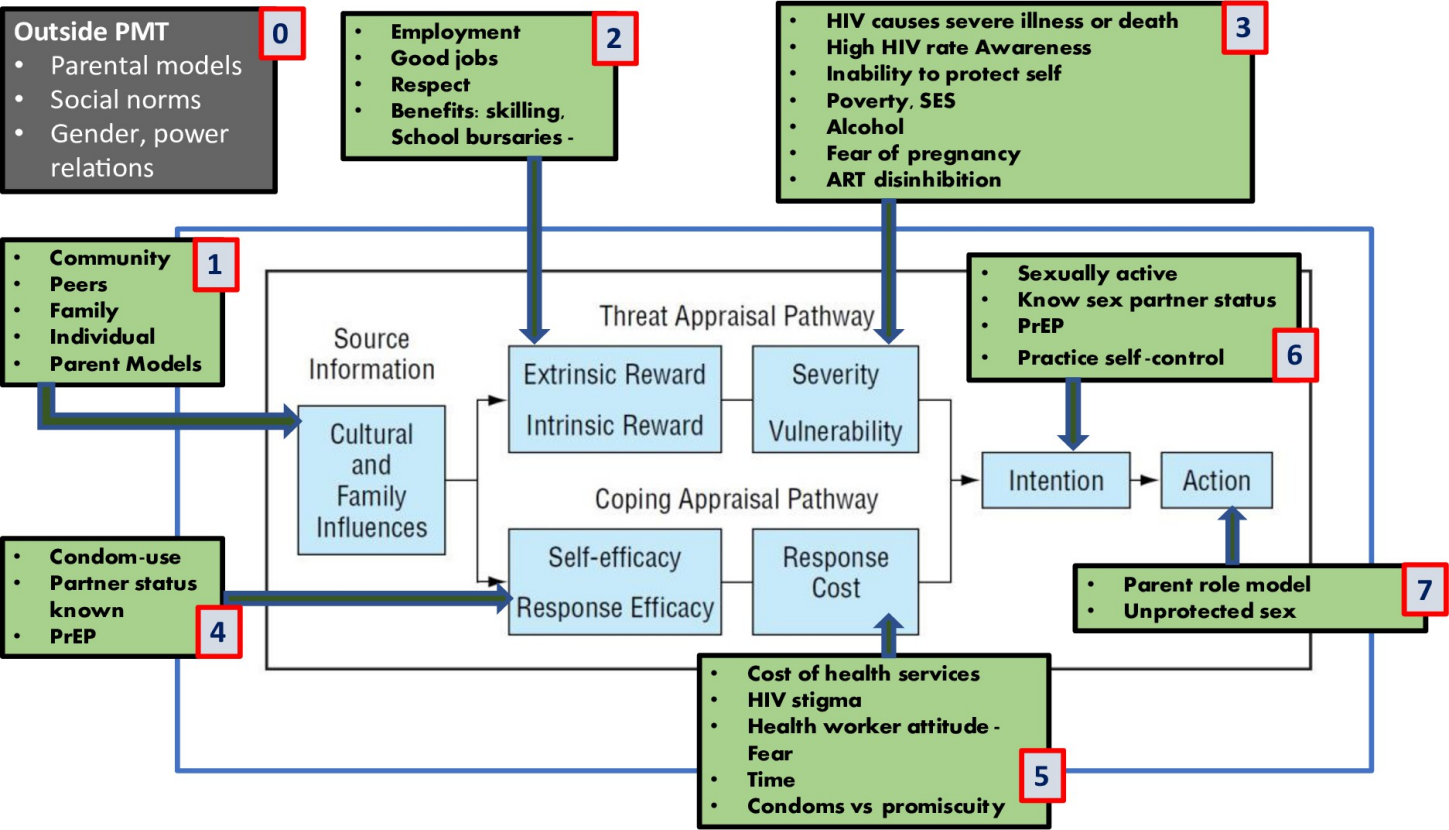
Contextualized Protection Motivation Theory (PMT) adolescent girls and young women. Key: Green box = data-driven themes, Blue box = PMT, Grey/black box = Additional themes identified.

*Source of information*. From 34 FGD with AGYW and parents and 25 AGYW key informant interviews, we identified four main sources of influence for HIV risk behaviors for AGYW: family, community, parent/guardian, and peers. Peer influences were strong motivators leading to intention and actions of AGYW (Fig 1 and Box #1). The influence of parents and guardians on HIV risk behaviors came from both guidance and role modelling (Table 3 QO2). Most AGYW felt positively about the protective behaviors recommended and learned from either their family, community, or from exposure to HIV programs.

**Table 3:** Key themes of relevant PMT constructs from the AGYW HIV risk data analysis.

*Topic Area*	*Themes*	*Selected Quotes (Q denotes Quote number)*
***0. Outside PMT***	Gender power imbalance *Cultural Norms* Parenting role model	*QO1. I don’t think a woman can stand on her own and tell the man to carry condoms. He is the one to decide and me if I was born a man I would not acquire HIV because is the one who controls his thing. And I want to say that as women we get HIV from men*. *(FGD 8-Par of Positive, Urban)* *QO2. In addition, as parents we also put our children at risk of getting infected with HIV*. *If parents do have sex openly before children, the child will also practice the same thing, and this puts her at risk of HIV (FGD 15, rural, Neg Par)*
***1. Source of Information***	Community Peers, Family, Parent/guardian	*Q1…*. *At times these girls have not seen successful people in their communities or families who have gotten degrees or diplomas. So they cannot go to a higher level. For example children from the slum areas. Families with members who have studied and succeeded with degrees can act as role models to the young girls and young women (FGD 11, Par, Urban)* *Q2*. *So it should be your role or duty as a mature person to tell this girl to be patient until when that time comes. Good things are in ahead. I know of a parent who called to have a word to an adolescent girl who had told her mum that she doesn’t want to work, no going to school but she wanted to go for marriage. Her mum told me that she had talked to the girl and got tired. (FGD 11, Par, Urban)*
***2. A-Rewards -Intrinsic -***	Future Employment Respect	*Q3*. *If a child is at school, she is protected from getting engaged with men and spends most of her time at school. Therefore, she will not have time for sex with men (FGD 15-Parents/guardian AGYW HIV negative, Rural)* *Q.4*. *I am of the view that both the boys and the girls should go to school because in the current economy we buy in the same market, when it comes to jobs now the women can do the jobs that men also do. Really I don’t support the view that it is more important for boys to go to school than girls. (KII 255 Pos, 19 years)* *Q5*. *The more you study up to a higher level, the more you get a respectful job…But for a girl if you don’t work as a house girl, like for me I fry cassava……Those are the things that you can do. Like working in the market. Such hard jobs (KII 294, 22years Neg)*
***2. B-Rewards- Extrinsic–external***	Good partner/husband Good job	*Q6*. *Education is important to our children because it will lead them to a good life which they will enjoy in their future (FGD 16, Parents -Neg)* *Q7*. *When you attend school, you get an office job or job in a shop because you have knowledge but a person who hasn’t gone to school will continue digging or working as a house girl (KII 5, 16 years AG)* *Q8*. *When you stick to education, you can get a good job (KII 292, AG-15 years)*
**3. A- Vulnerability**	*Material things (immediate against future aspirations)* Impact of Poverty Alcohol High HIV rates Inability to protect self Sexual exploitation Early marriage Teen pregnancy Peer pressure	*Q9. The young adolescent girls and young women yearn for material benefits which puts them at risk of getting infected with HIV. (KII 92 19 years Neg)* *Q10. Another reason is that a woman may be forced to have sex when she raped. (KII 078 Positive 17 years)* Q11. The rich old men force the young women to have sex because they have the money and the young women need money. (KII 92, 19 years Neg) *Q13. Some schools demand a lot of money and the parent cannot afford such an amount of money Even if he is working, it may be too much for him to pay such an amount of money. Sometimes these peer groups (KII 260 17 years, Neg)* *Q14. Here in Kampala if you don’t try, you can’t get what to eat, for me I get some place where to stand and then sell my body…..when it is available I can get like forty thousand shillings (KII 290, Pos, 23years)* *Q15. It’s because young girls get pregnant at an early age. Yet when a boy gets a girl pregnant, he can leave the child with someone to take of them and proceed with school unlike a girl. (KII 9, Positive, 16 years)* *Q16. Peer group influence also puts adolescent girls at risk of getting infected with HIV. If an adolescent girl involved in doing something, her colleague will also do the same thing. If one of the girls does have a mobile phone, another friend will also herself think why not hold a mobile phone as well.(KII 155, Negative 19 years)* *Q17. Its girls in school because a girl may be moving a long distance to school and then she finds a boda man who tells her that he will be taking her to school for free every day. To and fro. So a girl will think to herself, “oh the journey to school is so long, but this man will be able to help me go to and fro every day,” then the man will take her on day one without saying anything, he will always converse with her. (FGD 9, AGYW HIV Positive, Rural)* *Q18. Each small bag of alcohol is sold at seven hundred shillings [$0.13]. Now the price was reduced to five hundred shillings [$0.18]. So, a girl gets four thousand shillings ($0.11) and eight bags are enough to get her drunk. Then you find her fallen on the roadside and whichever man wants is the one who use her for sex (FGD 11 AGYW HIV Pos, Rural)* *Q19. …peer group influence was the major reason why I had sex with him. This group includes my friends or my fellow students here at school in form three (senior three). All these friends had their guys or boyfriends and they had sex with their boyfriends sometime back. … my friends talked explained to me about things that happen while playing sex. As our conversation continued, one of my friends asked, “have you had sex before?” I replied no. They also explained that if you have not had chance to have sex with your boyfriend, it implies that you don’t love him. Okay, this was the kind of message I got from my friend. So, I picked interest to have sex with my boyfriend. (KII 2- HIV negative, 17 years, Rural)*
***3. B-Severity***	Death, sickness Prevalence rates Fear of pregnancy than HIV Disinhibition	*Q20. Another reason is that a woman may be forced to have sex when she was raped. (KII 078 Positive 17 years)* *Q21. Do you know that most of the girls now fear pregnancy more than AIDS? AIDS right now is seen as malaria because they get good food and medicine and they even look better than the person who doesn’t have it……“instead of getting pregnant I would rather get AIDS” because they know that they will be taken care of. (FGD 4, 18-24years Neg, Urban)* *Q22. The reason why I think HIV is the biggest problem is that a woman may not test for HIV but may be pregnant. She delivers her baby in the village and the baby gets the HIV virus from her. You find that the baby and mother are both on HIV treatment (FGD 11- Positive Girls 18–24)* *Q23. I heard on the radio that Bukomansimbi is the place with the highest number of children with HIV (FGD#9-AGYW HIV Positive-In school)* *Q24. I have pointed out HIV burden at 100 percent because adolescent and women constitute the biggest number. In fact, it looks like every young woman “amaaso agatwalidde mungalo (translated as carelessly having sex with anyone…).”(FGD 15, Neg Parents, Rural)* *Q25. Yes, it is a big problem because you are ever worried and thinking of the day when you are going to die (FGD 4 AGYW 18–24 Neg in school, Urban)* *Q26. “I can say that today HIV is like any other disease such as malaria, headache or fever. If I get infected unknowingly, If I get infected with HIV, I can go and get medication without my relatives knowing but with pregnancy my mother can even chase me away from home. (FGD 4, 18-24years Neg, Urban”)* *Q27 I am of the view that those women who carry condoms are not married and fear to get pregnant. So they engage in sex with many men. (FGD 5, Neg Parents, Urban)*
***4. Self-efficacy***	*Condom use* *Partner status known* *PrEP*	*Q28. PrEP is something which prevents HIV from entering the body (another one) they are tablets which you take everyday (FGD 1, AGYW, Neg Rural)* *Q29. You must use a condom to avoid getting infected with HIV or using a condom as family planning method/ contraceptive (FGD 1 AGYW Neg)* *Q30. Very few girls are educated and for instance if she is married at a young age, she will not ask her husband or boyfriend to go for a blood test. Out of one hundred girls, only ten of them ask their partners to go for the HIV test. They are affected. They get HIV before having children and this may hinder them from having children. (FGD 13 Negative parents 15–17)* *Q31.We are always told that condoms are bad to use. It is one of the dangerous family planning methods because we are told that condoms have chemicals. If these chemicals enter your body, it causes an effect on the uterus and you will ever have children completely. Therefore, this young woman should not use this condom. If she happens to get pregnant, she should wait until she gives birth to her baby. It should be noted that abortion is not right because you may die in the process of making abortion. (KII 2- HIV negative, 17 years, Rural)*
***5. Response cost***	*Cost of health services* *HIV stigma* *Health worker attitude- Fear* *Time* *Perception of Condom-use associated with promiscuity*	*Q32. I don’t know because now days health services are now paid for. So to be served well you have to pay money. (KII 082 Postive 16 years)* *Q33. There are some health workers whom you just look at and fear them. There are some health workers who are tough and make us fear them. This makes us fear coming back to them (FGD #3 AGYW HIV Negative, Rural)* *Q34. It is not true. I may come here at the health center with my handbag and pick some condoms. I may reach home and forget to remove the condoms from the handbag and get a sudden trip and just pick the handbag and leave. When someone checks my bag and find the condoms, she or he may think I have sex with a lot of men and yet it is not true (FGD 11, HIV Pos AGYW Rural).* *Q35. She will come to test and there are some VHTs or counselors at the facility. The health workers test her and find her HIV positive. The health worker then asks the counselor to go counsel the girl and make home visits to ensure that she starts HIV medicine. The counselor then starts talking about the girl that she has HIV on the entire village. This is the major thing that scares them from coming to the health facility. The counselors and VHTs are not confidential. They do not know that we can have HIV and live a long life and have children who do not have HIV. I can live for a long period of time longer than for a person who considers herself HIV negative (FGD #11 AGYW HIV Positive, Rural)* *Q36. Women who carry condoms are sex workers. They carry condoms expecting to have sex with men. (FGD 8-Parent Positive (15–17), Urban)* *Q37. Women moving with many condoms that is a sign of prostitution. Because if not why should you move with condoms? (FGD 9, Parent Negative, Urban)*
***6. Intention***	Sexually active Knowledge of sex partner status PrEP Practice self-control	*Q38. My submission is that, if you want to have sex, first test with that person you want to have sex with (FGD 4 -AGYW HIV Negative, Rural)* *Q39. You must use a condom to avoid getting infected with HIV or using a condom as family planning method/ contraceptive (FGD 1)* *Q40. There is also self-control where you avoid getting exposed to risky sexual behaviors like having sex anyhow (FGD 4)* *Q41. That is why we take PrEP because (another one) commercial sex workers are the most people who are at risk of acquiring HIV. I might have taken alcohol, get a man, but in the process of having sex he may take off the condom when I am not aware. He might be infected but this PrEP prevents me from getting HIV. (FGD 1-AGYW Neg)* *Q42. They test the partners they are going to have sex with. If the situation is urgent then use condoms (FGD#18-Parents/Guardians HIV Positive Rural)*
***7. Action***	Intimacy and condom-use	*Q43. We used a condom at the very beginning and the second time we did not use a condom. He told me that he loves me so much*. *So, there was no reason of using a condom. (KII 082-AGYW (18–24) Positive, Urban)*

*Threat appraisal pathway*. The AGYW perceived ‘education’ and ‘spending time on school’ as leading to future gains, especially employability, respect, and self-confidence (Fig 1 and Box #2). These perceived gains empowered AGYW and were fundamental factors in their willingness to adopt HIV prevention behaviors. Participants’ assessment of their HIV acquisition risk, particularly those seen as more ‘extreme,’ such as perception of the severity of HIV in the community, played a major role in their willingness to engage in protective behaviors. AGYW who perceived that early sexual activity interfered with education prospects were generally highly motivated to engage in positive behaviors around abstinence, condom use, or reducing sexual partners, because these behaviors helped avert the risk of early pregnancy. Participants perceived that education helped them stay busy and avoid some high-risk behaviors that idle, out-of-school youth sometimes engaged in (e.g., alcohol use and sex with multiple partners). Social norms around virginity increasing a girl’s desirability as a marriage partner was a strong facilitator to reducing early sexual debut. Participants reported that knowledge would prevent the acquisition of diseases like HIV or sexually transmitted diseases (Table 3, Q3, Q4, Q28).

The perception of the current risk of HIV acquisition was a crucial factor for some participants to avoid risky behaviors (Fig 1 and Box #3). Data demonstrated that many parents and girls viewed teen pregnancy as a higher risk than HIV among AGYW in Uganda (Table 3 Q21). Some participants were concerned about the high proportion of AGYW at risk of HIV infection. This was influenced by the number of young girls witnessed at the health centers on adolescent days and further discussed in the media (Table 3 Q23, Q34, Q35). The perceived inability and lack of confidence of adolescent girls to protect themselves from HIV were felt to be a significant risk. Young women generally perceived an inability to negotiate protective behaviors, including condom use and knowing their partner’s HIV status (Table 3-Q29).

Many participants perceived HIV to be associated with a high risk of severe illness or death if left untreated (Fig 1 and Box #3). There were also concerns that healthcare workers may not keep their HIV status confidential, increasing their fear of experiencing HIV-related stigma. However, AGYW also recognized that HIV treatment was widely available and that they could potentially hide their HIV status from their friends and family members if needed. In contrast, AGYW were more concerned about getting pregnant, which they would not be able to hide. This fear of pregnancy appeared to be a stronger motivating factor for adopting protective behaviors than fear of acquiring HIV (Table 3 Q21). Parents also further confirmed this fear of pregnancy (Table 3 Q27).

Some AGYW cited that economic status negatively affected their ability to prevent the acquisition of HIV. Hardship in attending school was particularly singled out as a key barrier that exposed AGYW to risky behaviors leading to HIV infection. For example, the inability of families to pay school fees was the main factor that promoted AGYW to drop out of school. In addition, harmful discipline methods like corporal punishment have affected the attendance levels for AGYW (Table 3 Q21). Collectively, these factors may lead to premature school dropouts and early sexual debut, which increase the risk for HIV infection.

Many participants presented peer pressure as a significant influence on the behavior of AGYW. Peer pressure was coupled with admiration of property and personal items that the girls could not afford. This desire for material possessions made them vulnerable to sexual exploitation by an older sex partner or to entering the sex trade, which increased their risk for HIV acquisition (Table 3, Q9, Q11, Q12).

Lastly, the effects of alcohol consumption among AGYW were identified as a key negative attribute, and there was some concern about the effect on mental well-being. Participants recounted several examples of girls engaging in high-risk sexual behavior while under the influence of alcohol. Alcohol use by young women was further associated with putting girls at risk for sexual abuse, and assault and domestic violence by partners and family members (Table 3, Q18). Alcohol was perceived as easily accessible with no restrictions to minors and inexpensive, making it dangerous for the daily wellbeing of AGYW, and a barrier to HIV prevention.

*Coping and appraisal pathway*. Most participants knew how to use condoms and understood their importance as a tool for preventing both pregnancy and HIV acquisition (Fig 1 and Box #4). However, few reported using condoms consistently, including AGYW living with HIV who reported partner refusal as a barrier, that they did not use condoms with partners who were either HIV-negative or of unknown status. Hence, the main factor hindering condom use was a perceived inability to negotiate condom use with their male partners (Table 3, Q42).

Some participants also feared that condom use would be perceived by their partners and families as an indicator of their promiscuity, also confirmed by discussions with parents (Table 3, Q27, Q34, Q37). In addition, AGYW cited negative attitudes of healthcare workers as a barrier to health seeking behaviors of young girls (Table 3, Q35). Most participants were knowledgeable about their partner’s HIV status with many reporting they had tested together.

While most AGYW were unaware of PrEP (pre-exposure prophylaxis), a few did mention PrEP as another HIV prevention strategy. One of the participants was currently receiving PrEP and was able to explain how it worked and the importance of using PrEP to prevent HIV infection. She emphasized her belief that PrEP was particularly important for people who were unaware of their partner’s HIV status and/or were unable to use condoms. Some AGYW also appeared to confuse PrEP with post-exposure prophylaxis.

Several costs were perceived by AGYW that hindered their ability to adopt HIV protective behaviors. Many AGYW expressed fear of accessing health services due to healthcare worker attitudes that stigmatized young girls attempting to access sexual and reproductive health services (Fig 1 and Box #5). While HIV treatment services are free in Uganda, AGYW often pay a small fee to access family planning and other sexual and reproductive health services. This payment can act as a barrier to accessing these services. AGYW also face difficulty affording the transport costs to seek facility services. Inconvenient clinic hours were a barrier, with some AGYW mentioning school vacation times as the only time they had available to access health services.

*Intentions and action*: *Reported behavior of AGYW in relation to HIV prevention*. Some participants were aware of programs that could support AGYW and reinforce positive behaviors to prevent HIV acquisition. This includes the DREAMS program, which was available in the rural community. In addition, participants described the ability to receive an HIV test and life skills education as part of the DREAMS program (Fig 1 and Box #6).

Access to HIV prevention programs for AGYW was an important factor in how feasible it was for AGYW to access HIV testing and condoms for protection. AGYW generally perceived these programs as attractive for the added advantage of skill building for self-development. Some AGYW worried about health worker attitude and HIV status disclosure from attending health facilities, citing stigma and concerns about healthcare workers potentially violating confidentiality (Table 3 Q33, Q35). Despite the high knowledge of condom use and access, very low or inconsistent condom use was reported among HIV-infected and un-infected AGYW. Most girls associated trust and love with non-condom use (Fig 1 and Box #7), irrespective of partner’s HIV status (Table 3, Q42).

*Constructs outside the PMT*. We identified themes relevant for protective behavior among young women which were not part of the original PMT. One such theme depicting gender imbalance and power relations (Fig 1 and Box #0) explained AGYW’s reported inability making safe healthy decisions by themselves, driven by a norm that gave men power over women as illustrated in Table 3, QO1.

Secondly, the importance of parenting and role modeling through perceived effectiveness of behaviors was communicated through knowledge and the willingness to perform the behaviors. (Fig 1 and Box#0) Parents and guardians further reinforced their influence in modelling the protective behavior that would be important for young women to learn from them (Table 3, QO2).

Lastly, the effect of social norms affected motivation to engage in certain behaviors. The view that girls and men having children before marriage related to HIV acquisition risk, with varied range of perspectives on when a girl should be ready to have children.

## Discussion

Our findings highlight complex multidimensional pathways including poverty, gender inequality, parental behavior and social norms affecting HIV acquisition. AGYW were knowledgeable about most HIV prevention interventions though less familiar with PrEP. Many AGYW thought access to these programs would help facilitate healthy, sex-positive, protective behaviors. Alcohol use, desire for material possession, incorrect perception of HIV disease severity, social norms, and poverty were barriers to self-protective behaviors. While condom knowledge and access were high, usage remained low, resulting from structural barriers, negative community perceptions, and associations with promiscuity [22]. Conservative perceptions around condom use and promiscuity have long hindered and increased stigma around condom use while the scientific literature demonstrates overwhelmingly that social conservatives’ promiscuity argument on sex education is false [23]. From large-scale systematic reviews from the 1970s to the 1990s evidence consistently indicate the value of sex education with no links to increased sexual activity in young people. Further, understanding the socio-economic complexities driving sex, for basic needs, improved social status and material expressions of love need to be accommodated to best design successful prevention programs [24, 25]. Additionally, some AGYW and parents showed higher risk perception for pregnancy than HIV seemingly generated from cultural and social norms. This is essential for programs to address. Studies generally indicate that young people, especially adolescents, are more inclined to worry about the current and immediately visible impact of unprotected sexual behavior, such as unplanned pregnancies, rather than the behavior’s long-term consequences [26]. Low HIV risk perception may additionally be explained by existing HIV treatment optimism (i.e. confidence into the availability and effectiveness of HIV treatment), although evidence on this causal explanation seems to be inconclusive [26–28]. With sexual debut for Uganda reported at about 33–40% below age 19, this underscores the importance of addressing challenges faced by young people. This calls for comprehensive interventions for the broader HIV prevention and Sexual and Reproductive Health (SRH) rights context [29]. Our analysis corroborates other study findings on condom-use gaps among HIV-infected and non-infected AGYW associating non-use with “trust/love,” meriting creative, intentional condom messaging for boys and couples that targets the ability of girls to negotiate condom use [22, 30, 31]. Deliberate strategies that improve risk perception of both female and male condoms while responding to access and acceptability concerns around the female condoms would be key for condom-use uptake [32].

Many AGYW viewed staying in school as an important protective factor regarding preventing risky behaviors during schooling and later in life. Education is further critical for accurate risk perception of HIV among AGYW [33]. Furthermore, schools can lead to the economic empowerment of AGYW, an important factor associated with protecting AGYW against HIV acquisition [34, 35]. Programs should strive to work with communities to ensure that AGYW are educated, offering educational subsidies appropriately and job training to empower them to make their own choices and negotiate safe sex [4, 36]

Evidence [26, 28] does show that materialism, including a desire for glamour and luxury can become an important motivation to engage in sexual relationship for adolescent girls and young women in the sub-Saharan African region. Having certain material goods may be deemed essential to avoid social exclusion especially for adolescent girls and young women, for whom belonging to and being acknowledged by the peer group is important for their social status [26]. This way, forming a socially acceptable identity may be intricately intertwined with their self–esteem, which in itself can be linked with vulnerability to sexual risk [28].

Research evidence suggests that this phenomenon is particularly prevalent in adolescence, therefore reducing transactional sex during this developmental phase is particularly important for HIV prevention [37].

Socio-cultural norms restricting sexuality communication impede open discussion of SRH matters between adolescents and young people and their parents [38]. Adolescent sexuality is often viewed as something that needs to be controlled and repressed, and thus sexuality communication between parents and adolescents in the sub-Saharan African region tends to be punitive, focusing on abstinence, rather than information about contraceptives and/or condoms [39]. This is particularly relevant in countries such as Uganda that have had a history of conservative SRH programming based on abstinence. However, recent sexuality education policies and guidelines in Uganda have started to recognize the need to act on unintended consequences of sexual behavior, such as unplanned pregnancies, thus being a step in the right direction. The socially driven norms around condom use perception related to promiscuity would require intentional targeted messaging at different levels.

Our previous research and other studies show that parents can be powerful protectors against adolescent risk behavior through mentoring, communication, and modeling [40]. Parents can provide guidance and model desired behavior. Youth cited parental influences as a key factor that can improve AGYW behavioral intentions. Parents also acknowledged the importance of serving as role models for their children regarding respectful behavior towards their spouse and safe, consensual sex. In addition, parents felt that being knowledgeable about the effectiveness of prevention interventions was important for them to make informed decisions about their children’s uptake of interventions. The PEPFAR DREAMS program promotes parenting programs, and Uganda implements Sinovuyu (teen parenting program) and other evidence-based programs like Families Matter! [4, 41] These programs educate parents about prevention interventions for AGYW and how to be positive role models to impact behavioral intentions among their children.

Gender and economic inequality emerged as a critical HIV risk factor for AGYW, with unequal power dynamics in sexual relationships and social expectations that obligate AGYW to provide sex in return for resources. This finding does not fit very well with the PMT and is thus worth considering as an important addition. Gender inequality negatively affects condom negotiation and self-efficacy, limits educational advancement, thereby affecting agency, limits gainful employment, and encourages violence within relationships [42]. Further, as is evidenced in research with young people in sub-Saharan Africa, the relation between gender roles and sexual agency is complex [43] Gendered community norms result in expectations around women’s autonomy, ideal or actual sexual network structures (e.g. concurrency or serial monogamy), gender power and material exchanges within relationships [44]. However, despite cultural shifts in gender-norms, the sexual agency of adolescent girls and young women in much of the African continent remains constrained by inequitable gendered power dynamics [45].

Targeted interventions addressing cultural and structural barriers to women’s advancement, including education, discrimination, harmful gender roles coupled with partner violence, could be developed to improve AGYW health.

Our findings identify alcohol as a barrier that accentuates risk for AGYW with limited programs focused on the impact of alcohol use [46]. Alcohol use during the transition from adolescence to young adulthood could reflect normative behavioral intentions from the young person’s lens. However, for many young people, drinking negatively affects their ability to control sexual and protection behavior [47]. Programs investing in understanding how alcohol use fits in young people’s lives, and how to build a stronger foundation to counter alcohol risk factors may avert the negative consequences. Too often, these factors affect mental health trajectories resulting from heavy drinking and other adverse socioeconomic effects on the social determinants of health for the current and future generations [46]. Understanding the impact of alcohol on a country’s burden of mortality as well as an economic, physical, and emotional disability could help increase investment in this area which is currently very minimal. Investments in substance use treatment for youth and structural interventions limiting alcohol availability may be worthwhile.

For HIV prevention among AGYW, the discussion among families and communities on the implications of short-term aspirations can be a cornerstone for success. Our data showed that ‘wanting of material things’ increases the risk of AGYW by negatively impacting safe sexual behavior and future HIV prevention efforts [48]. Programs could capitalize on interventions that focus on AGYW’s aspirations when addressing their sexual and reproductive health risks by finding innovative ways of engaging AGYW to focus on long-term goals for financial success [49, 50]. Working with AGYW and their community on intentional messaging could help address this behavior.

### Limitations

Our study uses self-reported data, with the potential for social desirability bias as participants tend to supply information thought to be more favorable or socially acceptable as opposed to being fully reflective of actual feelings or thoughts [33]. Data collected through FGDs may be overrepresented by some youths and underrepresented by others, although the trained facilitators encouraged all youth to give their opinions and emphasized that their opinion counted. Additionally, we did not interview male partners of AGYW due to concerns around possible inadvertent HIV status disclosure and other harms. Despite these limitations, the study brings several strengths, including theory-guided design, rigorous methods, and rich data, which produced important information for HIV prevention activities for AGYW and their male sexual partners. The degree to which the findings are not transferable to other groups of AGYW was attenuated by the diverse and purposively collected sample.

## Conclusions

AGYW have distinct HIV risk factors that suggest the need for policy and gender and economic equality improvement interventions, education opportunities, agency, and safe sex practices. While the PMT focuses on individual factors to promote behavior, our study findings reveal that HIV risk behavior is influenced by complex contextual factors like poverty, gender inequality and cultural norms. When applying individually centered theoretic behavioral models especially in low-income countries, they could benefit if contextualized through integrating locally appropriate structural constructs. Targeted adolescent-focused HIV interventions addressing violence, alcohol consumption, material wealth aspirations, health worker stigma, educational opportunities, and safe-sex practices and PrEP scale-up may prevent HIV in AGYW and facilitate HIV epidemic control.

## Supporting information

S1 Appendix Detailed characteristics of key informant interviews and focus group discussions. (DOCX)
