# Supplementary material for: Community and familial dynamics influencing risk behavior for HIV acquisition among adolescent girls and young women in Uganda: Qualitative analysis using Protective Motivation Theory
Source: PLoS One. 2025 Jan 24;20(1):e0301311. doi: 10.1371/journal.pone.0301311 (PMC11759986; doi:10.1371/journal.pone.0301311)
Supplement: S1 Appendix — (DOCX) [file pone.0301311.s001.docx]

S1 Appendix:

**Detailed characteristics of Key Informant Interviews and Focus Group Discussions**

| **Key Informant Interviews** | | **Age**  **15-17** | **Age**  **15-17** | **Age**  **18-24** | **Age**  **18-24** | **All** |
| --- | --- | --- | --- | --- | --- | --- |
|  | | **In School** | **Out of School** | **In School** | **Out of School** | **Totals** |
| Urban (Kampala) | HIV Negative | 3 | 1 | 2 | 2 | **8** |
|  | HIV Positive | 1 | 1 | 2 | 1 | **5** |
| Rural (Rakai) | HIV Negative | 1 | 3 | 1 | 2 | **7** |
|  | HIV Positive | 1 | 2 | 1 | 1 | **5** |
| **Total** |  | **6** | **7** | **6** | **6** | **25** |

| **FGDs** | | **Age group** | **HIV status** | **School Status** | **Number of participants** |
| --- | --- | --- | --- | --- | --- |
| Urban (Kampala) | #1 | YW | Neg | Out | 7 |
|  | #2 | YW | Neg | In | 9 |
|  | #3 | AG | Neg | In | 10 |
|  | #4 | YW | Neg | In | 10 |
|  | #5 | AG | Pos | In | 7 |
|  | #6 | AG | Pos | Out | 6 |
|  | #7 | AG | Pos | In | 11 |
|  | #8 | Par-AG | Pos | Out | 6 |
|  | #9 | Par-AG | Neg | Out | 10 |
|  | #10 | YW | Neg | In | 10 |
|  | #11 | AG | Neg | In | 10 |
|  | #12 | Par-AG | Neg | Out | 8 |
|  | #13 | AG | Neg | Out | 9 |
|  | #14 | AG | Neg | Out | 10 |
|  | #15 | Par-AG | Pos | In | 10 |
|  | #16 | YW | Pos | Out | 11 |
|  | #17 | YW | Pos | Out | 11 |
| **Total** | **17** |  |  |  | **155** |
| Rural (Rakai) | #1 | AG | Neg | In | 7 |
|  | #2 | AG | Neg | In | 6 |
|  | #3 | YW | Neg | In | 8 |
|  | #4 | AG | Neg | N/A | 7 |
|  | #5 | AG | Neg | Out | 6 |
|  | #6 | AG | Neg | Out | 6 |
|  | #7 | AGYW | Neg | Out | 6 |
|  | #8 | YW | Neg | Out | 7 |
|  | #9 | AGYW | Pos | In | 8 |
|  | #10 | AG | Pos | Out | 6 |
|  | #11 | YW | Pos | Out | 6 |
|  | #13 | Par-AG | Neg | In | 8 |
|  | #14 | Par-AG | Neg | In | 6 |
|  | #15 | Par-AG | Neg | Out | 8 |
|  | #17 | Par-AG | Pos | Mixed | 6 |
|  | #18 | Par-AG | Pos | Out | 8 |
|  | #19 | AG | Neg | In | 7 |
| **Total** | **17** |  |  |  | **116** |
